# Myocardial infarction patients referred to the primary care physician after 1‑year treatment according to a guideline-based protocol have a good prognosis

**DOI:** 10.1007/s12471-019-01316-w

**Published:** 2019-08-07

**Authors:** M. C. Bodde, N. E. van Hattem, R. Abou, B. J. A. Mertens, H. J. van Duijn, M. E. Numans, J. J. Bax, M. J. Schalij, J. W. Jukema

**Affiliations:** 1 Department of Cardiology, Leiden University Medical Center, Leiden, The Netherlands; 2 Department of Medical Statistics and Bioinformatics, Leiden University Medical Center, Leiden, The Netherlands; 3 General Practice H.J. van Duijn, Katwijk, The Netherlands; 4 Department of Public Health and Primary Care, Leiden University Medical Center, Leiden, The Netherlands

**Keywords:** STEMI, prognosis, general practitioners, practice guideline, left ventricular function

## Abstract

**Introduction:**

Identifying ST-elevation myocardial infarction (STEMI) patients who can be referred back to the general practitioner (GP) can improve patient-tailored care. However, the long-term prognosis of patients who are returned to the care of their GP is unknown. Therefore, the aim of this study was to assess the long-term prognosis of patients referred back to the GP after treatment in accordance with a 1-year institutional guideline-based protocol.

**Methods:**

All consecutive patients treated between February 2004 up to May 2013 who completed the 1‑year institutional MISSION! Myocardial Infarction (MI) follow-up and who were referred to the GP were evaluated. After 1 year of protocolised monitoring, asymptomatic patients with a left ventricular ejection fraction >45% on echocardiography were referred to the GP. Long-term prognosis was assessed with Kaplan-Meier curves and Cox proportional hazards analysis was used to identify independent predictors for 5‑year all-cause mortality and major adverse cardiovascular events (MACE).

**Results:**

In total, 922 STEMI patients were included in this study. Mean age was 61.6 ± 11.7 years and 74.4% were male. Median follow-up duration after the 1‑year MISSION! MI follow-up was 4.55 years (interquartile range [IQR] 2.28–5.00). The event-free survival was 93.2%. After multivariable analysis, age, not using an angiotensin-converting enzyme (ACE) inhibitor/angiotensin-II (AT2) antagonist and impaired left ventricular function remained statistically significant predictors for 5‑year all-cause mortality. Kaplan-Meier curves revealed that 80.3% remained event-free for MACE after 5 years. Multivariable predictors for MACE were current smoking and a mitral regurgitation grade ≥2.

**Conclusion:**

STEMI patients who are referred back to their GP have an excellent prognosis after being treated according to the 1‑year institutional MISSION! MI protocol.

## What’s new

- Selecting ST-elevation myocardial infarction (STEMI) patients who could be referred to the general practitioner (GP) can improve patient-tailored care.
- STEMI patients treated with pPCI and referred back to their GP have an excellent prognosis with a 5-year event-free survival rate for all-cause mortality of 93.2% after 1 year MISSION! MI follow-up.
- 4 out of 5 STEMI patients remained event-free for MACE after they were referred to the GP.
- Since there are no recommendations in international guidelines for the appropriate duration of follow-up in the outpatient clinic after a STEMI, this 1‑year period might be applied in future guidelines.

## Introduction

Due to the implementation of various very successful treatments for ST-elevation myocardial infarction (STEMI), such as treatment with primary percutaneous coronary intervention (pPCI), adjunctive antithrombotic therapy and adequate secondary prevention medication [1–6], the current 1‑year and 5‑year all-cause mortality rates in STEMI patients decreased over the last decades to approximately 10% [7, 8] and 20% [8, 9] respectively. In an era of growing economic pressure on healthcare, identifying low-risk STEMI patients can improve patient-tailored care and could reduce healthcare costs. For example, several studies demonstrated that low-risk STEMI patients can be safely discharged within two or three days after admission [10, 11], which resulted in a reduction of healthcare costs [10, 12]. However, to our knowledge, there are no recommendations as to the appropriate duration of follow-up in the outpatient clinic of a cardiologist after STEMI. In accordance with the MISSION! myocardial infarction (MI) protocol[13], after 1‑year follow-up, asymptomatic patients with a left ventricular ejection fraction (LVEF) >45% on echocardiography, are referred back to their general practitioner (GP). The hypothesis of this study is that these patients can be safely returned to the care of their GP after 1‑year MISSION! MI follow-up. As the long-term prognosis of STEMI patients referred to the GP is unknown, the aim of this study was to assess the prognosis of patients referred back to their GP after treatment according to the 1‑year institutional MISSION! MI protocol in the Leiden University Medical Center (LUMC).

## Methods

### Study population

All patients treated with a pPCI for STEMI in the LUMC are included in the prospective MISSION! MI registry [13]. For this current observational retrospective analysis we evaluated all consecutive patients treated between February 2004 up to May 2013 who, after completion of the 1‑year MISSION! MI follow-up were returned to the care of their GP. Patients who died during the first year after their index infarction, or patients who were transferred during admission to another hospital due to logistic reasons were not included in this analysis. Logistic reasons were lack of available space for patients to admit, patient’s preference or when patients were transferred back to the referring hospital after the pPCI. STEMI was defined as typical electrocardiographic changes (ST-elevation ≥0.2 mV in ≥2 contiguous leads in V_1_ through V_3_, ≥0.1 mV in other leads, or presumed new left bundle branch block) and a typical rise and fall of cardiac biomarkers accompanied by chest pain for at least 30 min [14]. Since the data did not contain any identifiers that could be traced back to the individual patient and was obtained for patient care, the Dutch Central Committee on Research involving Human Subjects (CCMO) permits the use of anonymous data without prior approval of an institutional review board. This study was conducted according to the declaration of Helsinki.

### Study procedure

The institutional, guideline-based MISSION! MI protocol is a standardised clinical framework which consists of a pre-hospital, in-hospital and an outpatient phase to optimise clinical decision making and treatment up to 1 year after the index event [13, 15, 16]. The MISSION! MI protocol is in accordance with the current STEMI guidelines and was adjusted when required [15, 16]. In the pre-hospital phase, a high-quality 12-lead electrocardiogram was obtained. If a STEMI was diagnosed, patients were treated by the paramedics with a loading dose of clopidogrel or prasugrel, aspirin, heparin and intravenous glycoprotein IIb/IIIa inhibitors, if appropriate. During the in-hospital phase patients were directly transferred to the catheterisation laboratory for pPCI according to the current guidelines. If no contraindications existed, β‑blockers, angiotensin-converting enzyme (ACE) inhibitors and statins were administered within 24 h of admission. Dual antiplatelet therapy was additionally prescribed, consisting of aspirin 100 mg daily for life and prasugrel 10 mg daily or clopidogrel 75 mg daily for 12 months, if appropriate. During the outpatient clinic phase, 4 clinic visits were scheduled and patients were treated in accordance with current guidelines to reach the secondary prevention targets. Furthermore, several functional tests, such as a stress echocardiography and Holter registration, were obtained and, if necessary, an intervention was performed. An important part of the outpatient clinic care was emphasis on the need for drug compliance, as well as the education about, and modification of, lifestyle behaviour (smoking cessation, healthy diet, exercise and weight management). Patients also participated in a professional cardiac rehabilitation programme as part of the routine care, in which they had a dietician, psychologist and social worker at their disposal, as this has been associated with better one-year outcome [13, 17]. After 1 year of intensive monitoring, patients were, by protocol, returned to the care of their GP if they were asymptomatic with a left ventricular ejection fraction (LVEF) >45%.

### Data acquisition/Clinical data

In accordance with to our protocol, all risk factors, clinical features and laboratory measurements were systematically collected for each individual MISSION! patient in EPD-VISION, using a unique study code. Echocardiographic images were attained from patients at rest in left lateral decubitus position using a commercially available system (Vivid 7 and E9, GE, Healthcare, Horten, Norway). Standard M‑mode and 2D (colour, pulsed and continuous wave Doppler) images were obtained from the parasternal (long- and short-axis) and apical views (long-axis, 2‑ and 4‑chamber), using 3.5-MHz or M5S transducers, and digitally stored for offline analysis (EchoPac BT13, GE Medical Systems, Horten, Norway). The LVEF, wall motion score index (WMSI) and the grade of mitral regurgitation (MR) were measured according to the current echocardiographic recommendations [18, 19]. Clinical follow-up data were prospectively collected in the electronical patient files by independent clinicians. Data from patients were gathered from either out-patient chart reviews or by telephone interview. Information on the vital status was obtained from the Dutch municipal personal records database. Cause of death was retrieved from the GP.

### Study endpoint

The primary endpoint of this study is all-cause mortality. The secondary endpoint is a combined endpoint of coronary revascularisation, recurrent MI, implantation of an implantable cardioverter defibrillator (ICD) or a pacemaker, hospitalisation due to heart failure, stroke and death. All these adverse events combined have been defined as major adverse cardiac events (MACE).

### Statistical analysis

Data are summarised as means with standard deviation in case of normally distributed or as median with interquartile ranges (IQR) in case of non-normally distributed data. Categorised data are shown as numbers with percentages. Univariable Cox proportional hazard regression models were used to assess the association of age, gender and pre-specified covariates, which are known associated variables in literature, with all-cause mortality or occurrence of time-dependent adverse events (MACE) in STEMI patients [1–3, 20–23]. Age, gender and other variables significant at *p* < 0.10 were entered into a multivariable Cox model to calibrate a combined prognostic index to predict either all-cause mortality or MACE [24]. Hazard ratios (HR) and 95% confidence intervals (CI) were calculated. To classify GP patients into either high- or low-risk groups based on these Cox regression models, we dichotomised the prognostic index using the median value. Stratified by these two groups, Kaplan-Meier curves were then used to estimate and verify survival expectations (time to either all-cause death or MACE). The log-rank test was calculated to compare the cumulative incidences of the endpoints between the 2 groups. All statistical tests were two-tailed, *p*-values <0.05 were considered statistically significant. Analyses were performed with SPSS 23.0 statistical analysis software (IBM, Armonk, NY, USA).

## Results

Between February 2004 up to May 2013, 2943 patients were admitted to the LUMC and treated with pPCI for STEMI. During the first year after their index infarction 206 (7.0%) patients died and 964 (32.8%) patients did not follow the institutional MISSION! MI protocol for logistical reasons. In total, 1773 (60.2%) patients completed their 1‑year follow-up according to the MISSION! MI protocol. Of these patients 851 (48%) received follow-up in the outpatient clinic of a cardiologist according to the MISSION! MI protocol.

**Fig. 1:**
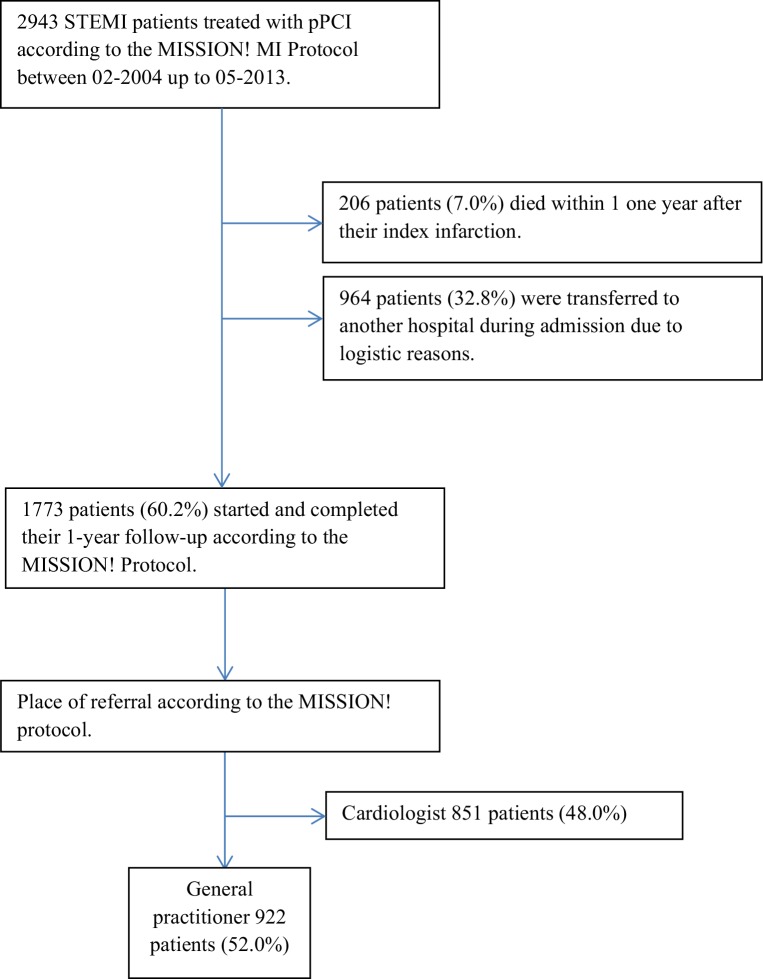
Overview of eligible MISSION! patients (*STEMI* ST-elevation myocardial infarction, *pPCI* primary percutaneous coronary intervention)

The other 922 (52%) patients were all referred back to their respective GPs and selected for evaluation (Fig. 1). Median follow-up duration after the 1‑year MISSION! MI follow-up was 4.55 year (IQR 2.28–5.00).

### Baseline characteristics

Patients characteristics, medication use and laboratory results after 1 year MISSION! MI follow-up are summarised in Tab. 1. Mean age was 61.6 ± 11.7 year and 686 (74.4%) was male gender. In 70 (7.6%) patients LVEF was below 45%.

**Table 1:** Patients characteristics after 1 year MISSION! follow up

Variable	GP (*n* = 922)
*Patient’s characteristics*	
Age, years	61.6 ± 11.7
Male gender	686 (74.4)
Current smoking	185 (20.1)
Diabetes mellitus	73 (7.9)
History of a malignancy	46 (5.0)
History of cerebrovascular disease	31 (3.3)
*Medication use*	
Beta blocker	824 (89.4)
ACE-inhibitor/AT2-antagonist	877 (95.1)
Statin	887 (96.2)
Aspirin	859 (93.1)
Coumarin	40 (4.3)
*Laboratory results*	
Total cholesterol (mmol/l)	4.14 ± 0.92
LDL-cholesterol (mmol/l)	2.39 ± 0.75
HDL-cholesterol (mmol/l)	1.34 ± 0.42
Triglycerides (mmol/l)	1.54 ± 0.82
*Echocardiographic parameters*	
Left ventricular ejection fraction <45%	70 (7.6)
Mitral regurgitation grade ≥2	31 (3.4)
Wall motion score index	1.13 (1.00–1.25)
*Clinical characteristics*	
Number of vessel disease during pPCI >1^a^	451 (48.9)
Complete revascularisation during pPCI	560 (60.7)
*Interventions*	
Revascularisation within 1 year FU	122 (13.2)

Data are expressed as number (%), mean ± standard deviation or median with interquartile range

*GP* general practitioner, *ACE* angiotensin-converting enzyme, *AT* angiotensin, *LDL* low-density lipoprotein, *HDL* high-density lipoprotein, *FU* follow-up, *pPCI* primary percutaneous coronary intervention

^a^A narrowed coronary artery was defined as a stenosis of ≥50% on baseline coronary angiogram

### Long-term survival analysis

In total, 48 patients deceased after 1 year MISSION! MI follow-up. The cause of death was adjudicated as cardiac origin in 6 patients, likely cardiac in 3 patients, non-cardiac in 35 patients, unlikely cardiac in 2 patients and the cause of death was unknown in 2 patients.

**Fig. 2:**
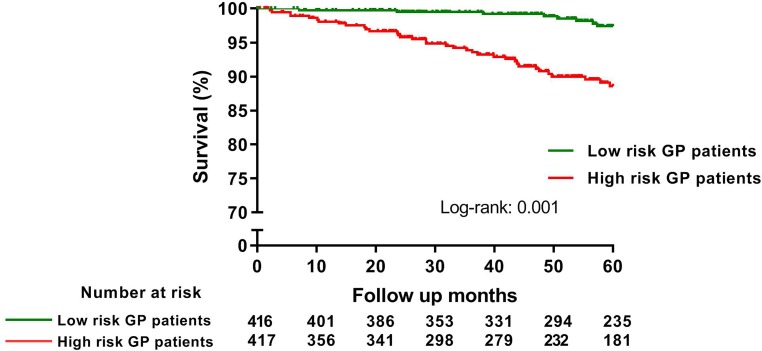
Kaplan-Meier analysis to evaluate the event-free survival of experiencing the primary endpoint of 5‑year all-cause mortality, stratified by high- and low-risk GP patients (*GP* general practitioner)

The event-free survival rate for the primary endpoint was 93.2% in the total GP group. Univariable Cox regression analysis revealed that age, history of a malignancy or stroke, not using an ACE-inhibitor/angiotensin-II (AT2) antagonist or aspirin, an impaired LVEF, an MR grade ≥2 and multivessel disease during pPCI were significant predictors for 5‑year all-cause mortality. After multivariable analysis, age, not using an ACE-inhibitor/AT2-antagonist and an impaired LVEF remained statistically significant predictors for the primary endpoint (Tab. 2). The GP patients were stratified by high risk and low risk. Fig. 2 shows that high-risk GP patients (*n* = 417) have a significant lower event-free survival rate of 88.6% compared with 97.4% in the low-risk GP group (*n* = 416) (log rank <0.001).

**Table 2:** Univariable and multivariable Cox proportional hazard regression analysis to identify independent predictors of 5‑year all-cause mortality

Parameter	Univariable analysis		Multivariable analysis	
	HR (95% CI)	*P*-value	HR (95% CI)	*P*-value
Age, y	1.085 (1.056–1.115)	<0.001	1.071 (1.040–1.108)	<0.001
Male gender	0.973 (0.506–1.870)	0.935	1.441 (0.678–3.064)	0.342
Current smoker	1.446 (0.757–2.764)	0.264		
Diabetes mellitus	1.725 (0.733–4.057)	0.212		
*Comorbidities*				
History of malignancy	2.812 (1.195–6.615)	0.018	1.896 (0.704–5.104)	0.205
History of cerebrovascular disease	3.359 (1.330–8.480)	0.010	1.077 (0.388–2.987)	0.887
*Current medication use*				
Beta blocker	0.493 (0.230–1.054)	0.065	0.498 (0.221–1.124)	0.093
ACE-inhibitor/AT2-antagonist	0.301 (0.119–0.760)	0.011	0.294 (0.110–0.788)	0.015
Statin	0.627 (0.152–2.586)	0.519		
Aspirin	0.424 (0.180–0.998)	0.049	0.831 (0.327–2.116)	0.698
Coumarin	2.002 (0.718–5.584)	0.185		
*Echocardiographic parameters*				
Left ventricular ejection fraction <45%	3.088 (1.493–6.388)	0.002	2.807 (1.298–6.071)	0.009
Mitral regurgitation grade ≥2	3.712 (1.465–9.406)	0.006	1.747 (0.642–4.755)	0.275
Wall motion score index	1.655 (0.638–4.349)	0.307		
*Clinical characteristics*				
Number of vessel disease during pPCI >1	2.043 (1.143–3.797)	0.017	1.540 (0.676–3.512)	0.304
Complete revascularisation during pPCI	0.585 (0.330–1.036)	0.066	1.041 (0.482–2.251)	0.918
*Intervention*				
Revascularisation within 1 year FU	1.302 (0.610–2.782)	0.501		

Data are expressed as hazard ratios with 95% confidence interval

*CHD* cardiac heart disease,* ACE* angiotensin-converting enzyme,* AT* angiotensin,* pPCI* primary percutaneous coronary intervention,* FU* follow-up

### Long-term MACE analysis

**Fig. 3:**
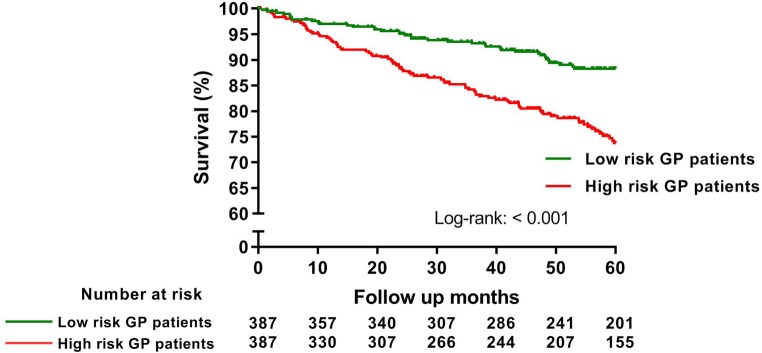
Kaplan-Meier analysis to evaluate the event-free survival of experiencing the secondary endpoint of MACE, stratified by high- and low-risk GP patients (*MACE* major adverse cardiovascular events, *GP* general practitioner)

In total, 147 reached the secondary endpoint. A recurrent MI occurred in 36 patients, in 51 cases a patient was revascularised, 42 patients died, 15 patients had a cerebrovascular event, in 2 patients an ICD or pacemaker was implanted and 1 patient was admitted for heart failure. In total, 80.2% remained event-free after 5 years for the secondary endpoint. Tab. 3 demonstrates the univariable and multivariable predictors for MACE within 5 years. Patients with an unfavourable outcome according to the univariate Cox regression analysis were patients who (1) were older; (2) had a current smoking status; (3) were not using aspirin; (4) had a lower LVEF; (5) had an MR grade ≥2; and (6) who had a multivessel disease during pPCI. Patients who underwent complete revascularisation during pPCI had a favourable outcome in the univariate analysis. Current smoking status and an MR grade ≥2 remained significant predictors for MACE after multivariable Cox regression analysis. Fig. 3showed the Kaplan-Meier curves of the patients who were stratified by high- and low risk GP patients. High-risk GP patients (*n* = 387) reached the secondary endpoint in 73.8% of cases, compared with 88.3% in the low-risk GP group (*n* = 387) (log-rank <0.001).

**Table 3:** Univariable and multivariable Cox proportional hazard regression analysis to identify independent predictors of 5‑year MACE

Parameter	Univariable analysis		Multivariable analysis	
	HR (95% CI)	*P*-value	HR (95% CI)	*P*-value
Age, y	1.016 (1.002–1.030)	0.029	1.008 (0.991–1.026)	0.370
Male gender	1.179 (0.797–1.745)	0.409	1.374 (0.862–2.189)	0.181
Current smoker	1.460 (1.010–2.109)	0.044	1.788 (1.190–2.687)	0.005
Diabetes mellitus	1.739 (0.683–4.432)	0.246		
*Comorbidities*				
History of malignancy	1.778 (0.985–3.210)	0.056	1.534 (0.765–3.074)	0.228
History of cerebrovascular disease	1.788 (0.911–3.510)	0.091	1.362 (0.639–2.902)	0.424
*Current medication use*				
Betablocker	0.830 (0.494–1.396)	0.483		
ACE-inhibitor/AT2-antagonist	0.659 (0.323–1.345)	0.252		
Statin	1.369 (0.436–4.296)	0.590		
Aspirin	0.529 (0.310–0.904)	0.020	0.381 (0.093–1.557)	0.179
Coumarin	1.757 (0.950–3.249)	0.073	0.562 (0.117–1.269)	0.471
*Echocardiographic parameters*				
Left ventricular ejection fraction <45%	1.987 (1.226–3.221)	0.005	1.649 (0.936–2.907)	0.083
Mitral regurgitation grade ≥2	2.759 (1.488–5.115)	0.001	2.463 (1.247–4.867)	0.009
Wall motion score index	0.870 (0.473–1.600)	0.654		
*Clinical characteristics*				
Number of vessel disease during pPCI >1	1.666 (1.194–2.325)	0.003	1.321 (0.794–2.197)	0.284
Complete revascularisation during pPCI	0.665 (0.478–0.926)	0.016	0.802 (0.490–1.314)	0.381
*Intervention*				
Revascularisation within 1 year FU	1.074 (0.677–1.704)	0.763		

Data are expressed as hazard ratios with 95% confidence interval

*CHD* cardiac heart disease,* ACE* angiotensin-converting enzyme,* AT* angiotensin,* pPCI* primary percutaneous coronary intervention,* FU* follow-up

## Discussion

In the present study, we demonstrated that STEMI patients treated with pPCI and referred back to the GP after being treated according to the MISSION! MI protocol, have an excellent prognosis with a 5-year event-free survival rate for all-cause mortality of 93.2%, after 1 year MISSION! MI protocol follow-up. Furthermore, 4 out of 5 patients remained event-free for MACE after they were referred to the GP. In an era of growing economic pressure on healthcare, identifying low-risk STEMI patients, can improve patient-tailored care and could reduce healthcare costs. Since there are no recommendations in international guidelines for the appropriate duration of follow-up in the outpatient clinic after a STEMI, this 1‑year period might be applied in future guidelines.

The decision whether STEMI patients can be returned to the care of their GP in the MISSION! MI protocol is mainly based on the LVEF measured after 1‑year MISSION! MI follow-up. An impaired left ventricular function in STEMI patients is strongly associated with worse outcome [21, 22], and an ejection fraction of 45% seems to be a good discriminator between high and low risk patients [21, 25].

There are several findings in this study that support the idea that patients can be referred to the GP after 1‑year MISSION! MI follow-up. First, in line with other large registry studies [7–9], this study shows that after the first year after STEMI, the yearly risk-of-death decreases. In the current analysis, the annual mortality rate was slightly more than 1% after 1 year MISSION! MI follow-up. Secondly, cardiac death was observed in a very small number of patients. In the majority of the patients, 37 out of 48 patients, the cause of death was of non-cardiac origin, mainly malignancies and pulmonary diseases, which is in line with results found by Pedersen et al. [8]. Furthermore, according to the Central Bureau of Statistics (CBS) in the Netherlands, the 5‑year survival rate for a 61-year-old healthy individual, which is the average age of the cohort referred to the GP, is 95.8% [26]. This is only slightly better than the observed risk of STEMI patients referred to the GP.

All patients in this study were treated according to the institutional MISSION! MI protocol. Other studies confirmed earlier that the extent of guideline implementation is associated with improved outcome [27–29]. The MISSION! MI protocol contains one structured patient-centred framework with a pre-hospital, in-hospital and an outpatient phase. An important part of the care in the outpatient phase is the emphasis on the need for drug compliance and the education about, and modification of, lifestyle behaviour. For example, a high percentage of patients still used their medication, prescribed during admission, after 1 year follow-up. Several studies indicated the importance of medication adherence that prevent cardiovascular disease in patients with an acute MI [30], which is associated with positive health outcomes [30]. Another possible explanation for the low event rate is the well-organised primary care in the Netherlands. In the region ‘Zuid-Holland Noord’ which also covers the Leiden region, the GPs use a uniform cardiovascular risk management (CVRM) care programme for all patients with cardiovascular disease [31]. In this programme, the patient’s routine follow-up is performed by nurse specialists in primary care who monitor patients risk factors and adjust if necessary.

Several risk factors for a worse outcome were identified during this study. As this uncontrolled, observational study reflects the situation in the daily practice minor protocol deviations can be expected. A small percentage of patients referred to the GP had an LVEF <45% or an MR grade ≥2. These patients were at higher risk of developing an adverse event. These results emphasize the importance of these patients staying in the outpatient clinic of a cardiologist where closer follow-up is available and where, for example, additional treatment, such as heart failure medication, can get started when indicated or potentially the need for cardiac resynchronisation therapy, ICD implantation or left ventricle reconstruction can be considered. Furthermore, current smoking and not using an ACE-inhibitor were identified as risk factors for the development of an adverse event, which most likely reflects a surrogate marker for overall healthy behaviour [30]. Before these patients are returned to the care of their GP, it is important that these issues are discussed with the treating GP.

There are several limitations that should be pointed out. First, since this is a retrospective observational single centre study, with patients treated according to the MISSION! MI protocol, it is difficult to expand these results to other hospitals or countries. Secondly, this study may have introduced bias since a substantial number of patients was referred to the referring hospital after treatment with a pPCI. However, these patients were not referred due to medical reasons but due to logistic reasons such as lack of available space for patients to admit or patient’s preference. So, a random cohort of patients was referred back to the referring hospital thereby preventing selection bias. Thirdly, next to the LVEF, the presence of symptoms was a criterium to keep patients in the outpatient clinic of the cardiologist. In this study, no detailed information about the patients’ symptoms was available. Although not unquestionable, it is unlikely that there is a large proportion of patients with serious complaints returned to the GP since the number of adverse events in the GP group in the first year after referral was 4.4%. And finally, this project did not focus on the 50% of the patients who stayed in the outpatient clinic of the cardiologist. Perhaps, amongst this group, there are patients who should be considered to be low-risk as well and who could be referred to the GP. Future research is needed to optimise and identify all the patients eligible for referral to the GP after being treated according to the 1‑year MISSION! MI protocol and should evaluate the possibilities the refer stable patients after a STEMI within one year to the GP as has already been suggested in 2005 by Boomsma et al. [32].

## Conclusion

In conclusion, STEMI patients who are returned to the care of their GP after 1‑year MISSION! MI follow-up have an excellent prognosis for 5‑year survival and have a low risk for MACE. Patients with an impaired left ventricular function or an MR grade ≥2 should be considered as higher-risk patients and are better off in the outpatient clinic of a cardiologist.
